# The Effects of Different Factors on the Behavior of Neural Stem Cells

**DOI:** 10.1155/2017/9497325

**Published:** 2017-11-20

**Authors:** Lixiang Huang, Gan Wang

**Affiliations:** Department of Chemistry and Biology, College of Science, National University of Defense Technology, Changsha, Hunan 410073, China

## Abstract

The repair of central nervous system (CNS) injury has been a worldwide problem in the biomedical field. How to reduce the damage to the CNS and promote the reconstruction of the damaged nervous system structure and function recovery has always been the concern of nerve tissue engineering. Multiple differentiation potentials of neural stem cell (NSC) determine the application value for the repair of the CNS injury. Thus, how to regulate the behavior of NSCs becomes the key to treating the CNS injury. So far, a large number of researchers have devoted themselves to searching for a better way to regulate the behavior of NSCs. This paper summarizes the effects of different factors on the behavior of NSCs in the past 10 years, especially on the proliferation and differentiation of NSCs. The final purpose of this review is to provide a more detailed theoretical basis for the clinical repair of the CNS injury by nerve tissue engineering.

## 1. Introduction

The central nervous system (CNS), consisting of the brain and the spinal cord, is the most important part of the nervous system. In the CNS, a large number of nerve cells get together to constitute different circuits or networks so that the CNS can transmit, store, and process information, generate all kinds of mental activities, and control animal behaviors. However, once the CNS is damaged, it is extremely difficult to be cured, which involves both internal and external causes. The internal cause is that the regenerative ability of nerve cells in the brain and the spinal cord is weak and the loss of cells cannot be supplemented by the proliferation of mature nerve cells. The external cause is the formation of the microenvironment that inhibits nerve regeneration in injured sites, including the production of inhibitory factors that inhibit the regeneration of axons, the lack of neurotrophic factors that promote cell regeneration, and the formation of glial scars that impede axonal passage. For the past few years, the continuous development and improvement of cell transplantation technique have provided the possibility for the repair of the CNS injury.

Neural stem cells (NSCs) that can self-renew and proliferate and have the potential to differentiate into neurons, astrocytes, and oligodendrocytes in the nervous system are not only mainly distributed in the ventricular zone (VZ) and subventricular zone (SVZ) but are also distributed in the hippocampus dentate gyrus of adult mammals, olfactory bulb, spinal cord, compartment, striatum essence, cerebellum, cerebral cortex, and other parts [1]. Based on the characteristics of self-renewal, proliferation, multilineage differentiation, low immunogenicity, and migration of transplanted NSCs *in vivo*, NSC has become a very promising cell type for the treatment of the CNS injury. One of the purposes of NSC transplantation is to replace or complement the missing or dysfunctional neurons in the CNS. In addition, NSCs can also promote neuranagenesis by continuous secretion of neurotrophic factors.

Scientific studies have demonstrated that it is not ideal to simply transplant NSCs into the injured area because the NSCs implanted are primarily transformed into astrocytes [2]. Consequently, to clarify the mechanism of the factors that regulate the behavior of NSCs is the key for us to repair the CNS injury successfully. This paper summarizes the effects of different factors (including physical factors, chemical factors, biological factors, and material factors) on the behavior of NSCs in the past decade and provides a more detailed theoretical basis for the clinical repair of the CNS injury by nerve tissue engineering.

## 2. Physical Factors

Under normal circumstances, most of the NSCs in the CNS are in a resting state, but all of them have the potential to differentiate. When subjected to endogenous and exogenous stimulations, these NSCs will be activated, showing different growth and differentiation behavior [3]. Here, we summarize the physical factors that regulate the fate of NSCs from the perspectives of physical stimulation and oxygen treatment.

### 2.1. Physical Stimulation

In recent years, plenty of studies have shown that external stimuli such as sound, light, electricity, magnetism, and acupuncture can induce NSCs to respond. The effects of low-intensity pulsed ultrasound (LIPUS) on the cell viability, proliferation, and neural differentiation of induced pluripotent stem cell-derived neural crest stem cells (iPSCs–NCSCs) have been reported. Under the different output intensities of LIPUS: 0, 100, 300, 500, 700, 900, 1100, 1300, and 1500 mW/cm^2^, the viability and proliferation of iPSCs–NCSCs were obviously enhanced after 2 days, and the genes of neurofilament (NF-M), *β*-tubulin III (Tuj1), S100*β*, and glial fibrillary acidic protein (GFAP) were upregulated after 4 days by LIPUS of 500 mW/cm^2^ [4].

Humans have been living together with radiation, such as cosmic radiation and ground radiation and man-made radiation. Computed tomography (CT) and X-ray are typically used in hospitals to examine patients, and we use mobile phones and computers every day. Thus, whether radiation affects the human body or cells has caused widespread global concern. Different doses of X-rays were used to irradiate the NSCs, and researchers found that NSCs could restore the abilities of proliferation and differentiation after a short stop of proliferation temporarily when irradiated at low doses, such as 1 Gy; conversely, NSCs exposed to relatively high doses (>5 Gy) lost these capabilities [5]. Over the past years, NSCs have been exposed to the radiation of radio-frequency electromagnetic field (RF-EMF) 900 MHz with specific absorption rate (SAR) = 2 W/kg [6], RF-EMF 1710 MHz with SAR = 1.5 W/kg [7], RF-EMF 1800-900 MHz with SAR = 1.6 W/kg [8] that confirmed the effects of RF-EMF on the proliferation and apoptosis of the NSCs. In general, these studies indicate that proliferation and differentiation of the NSCs depend on the source, intensity of radiation, and the duration of exposure.

The use of electrotherapy has a long history, and the past application of electrotherapy treatment of diseases was mostly empirical. With the development of the research on human bioelectricity, it has been found that bioelectric phenomena are widespread in organisms, which involve embryonic development and tissue repair after injury. A culture system with the functions of supporting stem cell growth and regulating the levels of electric current has been invented. The biphasic current stimulator chip with indium tin oxide (ITO) electrodes generated both positive and negative currents. The results showed that biphasic electrical currents (BECs) obviously promoted the proliferation and neuronal differentiation of NSCs [9]. Another experiment was conducted to study the effect of electric field on cell migration and differentiation. The 115 V/m direct current (DC) electric field increased the migration of neural precursor cells (NPCs) by approximately four times compared with the control group through the calcium-dependent mechanism and caused NPC membrane depolarization without breaking and also facilitated the differentiation of NPCs into neurons rather than glial cells [10].

The effects of magnetic field on the nervous system have been studied a lot so far. Results showed that moderate magnetic field could change the function of the CNS, the extremely low-frequency magnetic field could affect the survival and apoptosis of nerve cells, and the pulsed magnetic field could relieve pain in rats. Repeated transcranial magnetic stimulation (rTMS) is a new method for the treatment of many neurological disorders. NSCs and neural progenitor cells were subjected to the rTMS with 1 Hz and 30 Hz. After 2 weeks, compared with the blank control group, the formation of neurospheres was significantly increased with the rTMS; furthermore, the number of neurospheres in the 30 Hz stimulation group was the largest. There was no significant difference in the neuronal differentiation between the two stimulation groups, but both of them were increased by 1.5 times compared with the control group [11]. Although there are numerous studies in this area, the mechanism of the magnetic field on the specific regulation of NSCs is still not clear.

With the electromagnetic pulse being widely used in all walks of life, the impact of pulsed electromagnetic fields (PEMFs) on NSCs has attracted more and more attention. High-intensity PEMFs were applied to neonatal rats with 0.1 Hz and 0.5–10 tesla (T) [12]. The results showed that the survival rates of NSCs in the 3.0 T and 4.0 T groups were higher than those in other groups, indicating that the growth of NSCs was promoted. On the contrary, the 6.0 T, 8.0 T, and 10.0 T groups significantly inhibited the growth of NSCs. Thus, 4.0 T was the most favorable PEMF intensity for the growth of NSCs.

Acupuncture is a form of alternative medicine in which thin needles are inserted into the body. It is a common method of treatment in traditional Chinese medicine, which has been used to induce or activate the proliferation and differentiation of endogenous NSCs for the treatment of CNS injury with the advantages of safe source, no immune response, and no ethical problems. It has been reported that Sprague-Dawley (SD) rats were divided into the sham-operated group, model group, and acupuncture group, and acupuncture was performed daily at the specific acupuncture points of the brain traumatic rats. After 7 days, the number of nestin, neurofilament protein- (NF-) 200 and GFAP-positive cells was most in the acupuncture group [13]. Another similar experiment was conducted at different times to detect the marker proteins expressed by the three groups of NSCs [14]. The results showed that acupuncture was an effective method to promote nerve regeneration and functional recovery.

### 2.2. Oxygen Treatment

Oxygen is one of the indispensable conditions for cell survival, which participates in the tricarboxylic acid cycle to produce energy for cell growth, proliferation, and synthesis of various desired components. It is well known that oxygen concentration mediates many ischemic behaviors and cerebral ischemia can cause the activation of NSCs. The effects of oxygen on the proliferation and differentiation of NSCs have been studied [15]. Researchers studied the effects of 0, 1, 2, 3, 4, 10, and 20% oxygen concentrations on NSCs. They found that the proliferation rate and the proportion of neuronal differentiation of NSCs in 2% oxygen were the greatest and the number of dead cells gradually increased with the decrease of oxygen concentration. However, the duration of low oxygen pretreatment is still controversial [16]. To solve this problem, another team attempted to culture NSCs in 5%, 10%, and 20% oxygen for 72 hours and 120 hours. Finally, they reported that the optimum concentration and time of low oxygen pretreatment were 5% oxygen for 72 hours, which could maximize the proliferation and neuronal differentiation of NSCs [17].

NSCs were cultured in three mediums: hypoxic conditioned medium (HCM) with 1% and 4% oxygen and normoxic conditioned medium (NCM). The experimental results showed that 4% HCM could facilitate the proliferation of NSCs and the differentiation into neurons as much as possible. However, this result was inhibited after the addition of PI3K/AKT and JNK inhibitors. Ultimately, researchers summarized that 4% HCM promoted the proliferation and differentiation of NSCs might be mainly through the PI3K/AKT pathway [18]. These findings raise the possibility of curing CNS injury with the NSCs.

So far, researchers have investigated the effects of physical factors on the behavior of NSCs in a macroscopic manner without any specific regulatory mechanisms. Thus, using physical methods for the treatment of CNS injury still has a long way to go.

## 3. Chemical Factors

### 3.1. Drugs

Drug treatment is the most traditional method, and the history of which is almost as long as the history of mankind itself. Although great progress has been made in medicine, we still cannot use drugs to cure the CNS injury. At present, numerous researchers have attempted to transplant NSCs which are stimulated by drugs into the sites of CNS injury in order to make NSCs proliferate and differentiate into the desired direction.

#### 3.1.1. Chinese Herbal Medicine

After screening a number of Chinese herbal medicines and their active ingredients *in vitro* and *in vivo*, Chinese herbal medicine has been demonstrated to have various effects on NSCs in many aspects [19]. Salvianolic acid B is the most abundant and bioactive content of the salvianolic acids in danshen [20]. It was found that 20 *μ*g/mL of salvianolic acid B was favorable for the proliferation and neuronal differentiation of NSCs and the growth-promoting activity was associated with the number of NSCs in the G2/S phase [21]. Tetramethylpyrazine (TMP), an active element derived from traditional Chinese medicine Ligusticum Chuanxiong, is widely used for the treatment of ischemic stroke [22]. The proliferation and differentiation of NSCs treated with TMP under the hypoxia condition have been studied. The phosphorylation of extracellular signal-related protein kinase (ERK) and p38 in the mitogen-activated protein (MAP) kinase family were involved in these experiments [23]. In another study, NSCs were isolated from the hippocampus of neonatal 1 day rats; then, NSCs were cultured in different concentrations of epimedium flavonoids (EF) without growth factors. Researchers concluded that EF had neurotrophic factor-like function to promote NSC proliferation directly. On the other hand, compared with the 10% fetal bovine serum (FBS) control, EF significantly improved the level of neuron differentiation and migration of NSCs [24]. Studies have shown that panax notoginseng saponins (PNS) have functions of protecting brain tissue and antiaging. The result that 17.5 *μ*g/mL PNS was most conducive to the proliferation of NSCs has been confirmed [25]. Area density, optical density, and the numbers of nestin/BrdU, nestin/vimentin, and nestin/Tuj1 positive cells were significantly increased after oxygen glucose deprivation (OGD) and PNS were given, suggesting that PNS benefited the nerve regeneration in the brain ischemic injury. Polygala tenuifolia Willd is used as the treatment and prevention of dementia, and its main active ingredient is named tenuigenin. Under the influence of tenuigenin, more neurospheres were formed and the number of Tuj1-positive cells and GFAP-positive cells was significantly higher than that of the same volume of the phosphate-buffered solution (PBS) control group [26], which might be the underlying reason of the medicinal value of tenuigenin (seen in Table 1).

Gastrodiae rhizome [27], *Astragalus mongholicus* [28], Angelica [29], and other herbs are well-known, and many researchers have studied their effects on the proliferation and differentiation of NSCs. In conclusion, Chinese herbal medicine has shown great potential in inducing the proliferation and differentiation of NSCs. With the deepening of research, Chinese medicine scholars are expected to explore a new regulatory system for NSCs so that Chinese medicine can take a further step in stem cell research.

#### 3.1.2. Western Medicine

Under the background of the continuous emergence of new drugs and the substantial increase of medical level, the drug market for the treatment of CNS diseases has been growing rapidly. It has been demonstrated that ketamine could affect the proliferation and differentiation of NSCs *in vitro* [30]. Thereout, ketamine was injected into the subventricular zone (SVZ) of neonatal rats. The results demonstrated that ketamine could alter neurogenesis by inhibiting the proliferation of NSCs, preventing the differentiation into astrocytes and promoting the differentiation into neurons [31]. Sphingosine-1-phosphate (S1P) is a potent lipid mediator transducing intracellular signals, which regulates cells' biological behavior in the CNS [32, 33]. Fingolimod (FTY720), a structural analog of S1P, has been used to affect the brain-derived NSCs [34]. The research showed that the proliferation and migration of NSCs were promoted and the formation of astrocytes was increased instead of significant neuronal differentiation. Simvastatin is an essential antihypertensive drug for basic medical systems. The proliferation and neuronal differentiation of NSCs induced by simvastatin showed a long-term neurorestoration effect on the injured brain, which was mediated by the activation of the Notch signaling pathway [35, 36]. For this reason, simvastatin has become one potential treatment for patients with traumatic brain injuries (seen in Table 1).

Compared with traditional Chinese herbal medicine, western medicine is adopted for the purpose of symptomatic treatment and fast acting. However, it needs further exploration for radical cure and less side effects on chronic diseases and incurable diseases such as CNS injury.

### 3.2. Other Chemical Molecules

Recently, chemical molecules have been widely used to guide the biological function of NSCs and their different structures and properties have exhibited different effects on NSCs. An increase of evidence has suggested that hydrogen sulfide (H_2_S) could act as a novel neuromodulator to intervene in the advances in treating brain diseases. Sodium hydrosulfide (NaHS), the H_2_S donor, induced the proliferation of NSCs associated with extracellular signal-regulated kinase ERK1/2 and the neuronal differentiation associated with the expression of differentiation-related genes [37]. Epigallocatechin-3-gallate (EGCG), the major component of green tea polyphenols with antibacterial and antioxidant properties, is not toxic when it is less than 10 *μ*M. At this point, EGCG stimulated the proliferation of NSCs and the formation of neurospheres, and most importantly, neuronal differentiation was promoted by EGCG via the activation of the PI3K/AKT signaling pathway [38].

Although many chemical molecules had a role in promoting the proliferation and differentiation of NSCs, researchers also analyzed the effects of the chemical molecules that are harmful to humans in life on NSCs. Bisphenol-A (BPA), an endocrine disrupter commonly used as a surface coating for canned food, was fed daily to pregnant rats. Studies revealed that BPA significantly altered the expression of neurogenic genes and the Wnt signaling pathway genes. In other words, BPA impaired the proliferation and differentiation of NSCs via the Wnt/*β*-catenin signaling pathway [39]. As we all know, many unhealthy food contain saturated fatty acids (SFAs). Excessive intake of SFAs is the main cause of elevated cholesterol, secondary to atherosclerosis increasing the risk of coronary heart disease. Palmitic acid (PA) is one of the SFAs. The greater the dose of PA, the stronger the inhibition of NSC proliferation. PA was even cytotoxic at high concentrations. Besides, results also showed that PA promoted NSC differentiation into astrocytes by activating Stat3 and had little effect on neuronal differentiation [40]. Recently, 6-OH-PBDE-47, the metabolite of polybrominated diphenylether-47 (PBDE-47) used as a flame retardant [41], has been studied. Researchers found that 6-OH-PBDE-47 was more cytotoxic for adult NSCs than its parent compound and its inhibition of neurogenesis was associated with the inhibition of the ERK5 signaling pathway [42].

### 3.3. Chemical Functional Groups

Previous studies have demonstrated that surface chemistry was able to modulate cell-matrix adhesions [43] and chemical functional groups were capable of regulating the growth and differentiation of cells [44–46]. For these reasons, glass coverslips were modified by the hydroxyl (–OH), sulfonic (–SO_3_H), amino (–NH_2_), carboxyl (–COOH), mercapto (–SH), and methyl (–CH_3_) groups for culturing NSCs to study the effects of different functional groups on the adhesion, migration, and differentiation of NSCs [47]. On the –NH_2_ surface, the number of cells migrated from the neurospheres was the largest; conversely, the number on the –OH surface was the least. NSCs cultured on the –NH_2_ surface exhibited an increase on neuronal differentiation, while the –SO_3_H surface was more favorable for the differentiation of NSCs into oligodendrocytes. On the –COOH surface and the –SH surface, NSCs showed similar effects on migration and viability and tended to differentiate into glial cells. In addition, a great deal of astrocytes was observed on the –OH surface and the –CH_3_ surface. Hence, the chemical functional group-modified surface provides a reliable chemical method for the design of biomaterials for nerve tissue engineering.

From the years of research, it is clear that chemical factors such as drugs, other chemical molecules, and chemical functional groups can affect the growth of NSCs through some proliferation and differentiation-related genes or signal pathways and provide a theoretical and experimental basis for cell therapy of treating CNS diseases. However, there are still many problems that need to be overcome, such as the cytotoxicity, clinical application, and drug response.

## 4. Biological Factors

Up to now, modern neuroscience has been developed to study the structure and function of the nervous system at the biomolecular level to clarify the mechanism of neural activities. Previous studies have shown that both the physical factors and the chemical factors have a significant effect on the proliferation and differentiation of NSCs. Similarly, the use of growth factors, proteins, cells, and other biological factors can also regulate the behavior of NSCs.

### 4.1. Proteins

Proteins are the material basis of all life and are important parts of the body cells. Based on the functions of proteins, researchers have been trying to use proteins to regulate the growth and differentiation of NSCs to achieve the purpose of repairing the CNS injury.

#### 4.1.1. Neurotrophins and Growth Factors

The method based on various growth factors is still the most important way to regulate the proliferation and differentiation of NSCs. Neurotrophins and growth factors are typical biologically active molecules, as well as the essential substances in cell growth, which promote the growth, development, and integrity of neurons and glial cells. So far, brain-derived neurotrophic factor (BDNF), neurotrophin-3 (NT-3), nerve growth factor (NGF), and epidermal growth factor (EGF) [48] have been the most commonly used factors.

BDNF stimulated the proliferation of NSCs and significantly increased the differentiation of NSCs into neurons and oligodendrocytes; in addition, BDNF upregulated the expression of Wnt/*β*-catenin signaling molecules (Wnt1, *β*-catenin). Nevertheless, these promoting effects were blocked when the specific inhibitor of the Wnt signaling pathway IWR1 was added, indicating that BDNF acted on NSCs by triggering the Wnt/*β*-catenin signaling pathway [49]. Recently, the research about using BDNF for the treatment of Alzheimer's disease [50] has also been carried out. NT-3 transfecting bone marrow-derived NSCs (BM-NSCs) has been reported [51]. It was found that NT-3 promoted the proliferation and differentiation of BM-NSCs into cholinergic neurons and increased the level of acetylcholine (ACh) in the supernatant. Compared to this method, another team transduced NT-3 into the rat embryonic cortical NSCs [52]. The result showed that NT-3 was beneficial to the proliferation and neuronal differentiation of NSCs and greatly improved the survival rate of NSCs. NGF, the earliest discovered factor in the neurotrophic factors, has been studied the most thoroughly so far, with the dual biological function of providing neuronal nutrition and promoting synaptic growth. When NGF was added to the medium containing basic fibroblast growth factor (bFGF), the number of NSC proliferation was 17 times higher than that of the serum-free medium control group and 2.5 times higher than that of the treatment group with bFGF alone in the medium [53]. Now, researchers are increasingly concerned about the effects of multiple neurotrophic factors on NSCs. They explored the combination of NGF, BDNF, and bFGF to induce NSCs. After one week, the experimental results reflected that the proportion of differentiated neurons in the multifactor groups (bFGF + NGF, bFGF + BDNF, NGF + BDNF, and NGF + BDNF + bFGF) was significantly higher than that of the single-factor groups (NGF, BDNF, and bFGF) and the proportion of neurons was the highest in the NGF + BDNF + bFGF group. Besides, NSCs continued to proliferate over time in all groups [54].

The effects of neurotrophins and growth factors on the regulation of NSCs can accelerate the recovery of neurological function, promote the growth of neurons and dendrites, and provide the feasibility for the treatment of senile dementia, neurasthenia, and spinal cord injury.

#### 4.1.2. Other Proteins

The cell cycle which is closely connected with the development, proliferation, and differentiation of NSCs is controlled by the activation and inactivation of cell cycle-related proteins. Cyclin D1 causes cells to enter the S phase by forming a complex that inactivates pRb through the interaction with cyclin-dependent kinase 4 or 6 [55]. The knockdown of cyclin D1 resulted in the apoptosis of NSCs and inhibited the differentiation of NSCs into astrocytes with no effect on the neuronal differentiation [56]. Cell cycle-dependent kinases (Cdk) also play a key role in regulating cell cycle. After double knockout of Cdk2 and Cdk4 in mice, a phenomenon of ablation was observed between the intermediate zone and the cortical plate [57]. In addition, researchers found that the compensation of Cdk2 was the root cause of NSC proliferation and the double knockout NSCs tended to differentiate into neurons. Leucine-rich repeat and Ig domain-containing Nogo receptor interacting protein-1 (LINGO-1) is a nervous system-specific transmembrane protein. After 6 days of differentiation in LINGO-1 neutralized cultures, the number of neurons differentiated by NSCs increased three times, and the number of astrocytes had a slight increase. However, the neutralization of LINGO-1 did not significantly influence the total number of cells compared to the untreated control group [58]. The mental retardation-associated protein srGAP3 has been demonstrated that it could affect the morphology, behavior, and function of SHSY-5Y cell line [59] and was associated with mental retardation [60], long-term memory [61], and neurogenesis. The results showed that the viability and proliferation of NSCs decreased significantly when srGAP3 was knocked out (LV3-srGAP3 infection). After culturing in a differentiation medium 7 days, the number of nestin and *β*-tubulin III-positive cells in the srGAP3 knockdown group was more than that of the control group (LV3-NC infection) and the number of GFAP-positive cells decreased [62].

### 4.2. Cocultivation of Cells

In order to establish a culture system which is more similar to the environment *in vivo* so that cells can communicate with each other and support the growth mutually, cell coculture technique has been developed. Coculture system contains the following functions: inducing cells to differentiate into other types of cells; maintaining cell function and viability; and regulating cell proliferation. At present, cell cocultures have been extensively used in cell research.

Endothelial cells (ECs) are one of the most common cells cocultured with NSCs. It has been found that ECs stimulated the proliferation and differentiation of NSCs with vascular endothelial growth factor (VEGF), possibly by activating the Notch, Wnt, and Pten signaling pathways. The expression of the Notch signaling pathway-related genes (*notch2*, *numb*, *Hes1*, and *Psen1*), the Pten signaling pathway-related genes (*Pten*, *Akt1*, and *PIP3*), and the Wnt signaling pathway-related genes (*Wnt3a* and *β-catenin*) increased significantly in *in vitro* coculture conditions [63–65].

Recent data suggest that microglia are associated with neurogenesis. When activated by endotoxins, microglias inhibited neurogenesis *in vivo*. In contrast, microglia activated by cytokines interferon- (IFN-) g and interleukin- (IL-) 4 enhanced the neuronal differentiation [66]. Here, NSCs were cocultured with microglia collected from ischemic or excitotoxic injured brain. Microglia released mitogenic factors that promoted the proliferation of NSCs and NSCs differentiated into neurons and oligodendrocytes as soon as possible [67]. It is a known fact that astrocytes are one of the differentiation products of NSCs. Studying the effects of astrocytes on the behavior of NSCs has attracted the interest of researchers. An *in vivo* experiment was designed in which both astrocytes and NSCs were transplanted into the ischemic striatum of the transient middle cerebral artery occlusion (MCAO) rat model [68]. Definitively, cotransplantation resulted in a higher survival, proliferation, and neuronal differentiation of transplanted NSCs than that of NSCs transplanted alone.

Bone marrow-derived mesenchymal stem cells (BM-MSCs) have been widely used in tissue engineering. Consequently, researchers tried to coculture BM-MSCs with NSCs. The proliferation and neuronal differentiation of NSCs and high expression of various growth factors were induced by BM-MSCs, but the glial differentiation was inhibited. In addition, protecting NSCs against the neurotoxin 6-hydroxydopamine was another beneficial effect of BM-MSCs on NSCs [69]. In light of the above findings, the mechanism of neurogenesis induced by BM-MSCs was worth exploring. The result reflected that the enhancement of the proliferation and differentiation of NSCs was concerned with the upregulation of chemokine (C-C motif) ligand 2 (CCL2) released from BM-MSCs [70].

In addition to the large number of neuronal deaths, CNS injury is difficult to repair due to the regenerated axons without the ability to pass through the glial scar. Olfactory ensheathing cell (OEC) is a unique type of glial cells derived from the olfactory placode and occurs along the olfactory nerve in both the peripheral and central nervous system [71]. OECs could help the axons of neurons pass through the glial scar to promote functional recovery. Furthermore, the proliferation, neuronal differentiation of NSCs, and the formation of axons were promoted when NSCs were cocultured with OECs [72].

Cell coculture technique is a relatively safe, new, and promising technology in nerve tissue engineering. The interaction of supporting growth and promoting differentiation between the cells can be observed through the coculture system. At present, this technology has good applications in stem cells, tumor biology, and other aspects.

### 4.3. MicroRNAs

MicroRNAs are one sort of endogenous and noncoding RNAs that downregulate gene expression through the translational inhibition or degradation of their target mRNA [73]. The role of microRNAs on NSCs has attracted extensive attention of researchers. In NSCs, miR-34a, miR-125b, miR-146, miR-342-5p, miR-184, miR-9, miR-7, and miR-124 are important regulators (seen in Table 2).

MiR-34a, a member of the miR-34 family, is encoded by its own transcripts [74]. It has been reported that the overexpression of miR-34a increased the neuronal differentiation and neurite outgrowth of NSCs, which involved the downregulation of the silent information regulator 1 (SIRT1) and the enhancement of p53-DNA-binding activity [75]. Another regulator of neuronal differentiation, miR-125b, is highly expressed in the CNS [76]. MiR-125b inhibited the proliferation of NSCs and promoted the neuronal differentiation and migration by inhibiting its downstream target nestin [77], which is a necessary cellular process (proliferation, differentiation, and migration) regulator of NSCs [78, 79]. Similarly, miR-146, which is mainly involved in the regulation of inflammation and innate immune [80], is one of the brain-specific miRNAs. It had the same inhibitory effect on the proliferation of NSCs as miR-125b. NSCs tended to differentiate into glial cells rather than neurons by inhibiting the expression of Notch 1 with the overexpression of miR-146 [81]. The Notch signaling pathway negatively regulated miR-342-5p by its transcriptional repressor Hes5. Studies have shown that transfection of miR-342-5p induced the apoptosis of NSCs, whereas the differentiation of NSCs into intermediate progenitor cells (INPs) was promoted. Notably, the suppression of the differentiation into astrocytes was regulated by miR-342-5p directly targeting GFAP [82]. Methyl-CpG-binding protein 1 (MBD1) has been demonstrated that it was capable of controlling cell growth [83]. Accordingly, the study was conducted to indicate that the high expression of miR-184 regulated by MBD1 directly promoted the proliferation of NSCs and inhibited their differentiation [84]. Numb-like protein (Numbl), which was the downstream target of miR-184, MBD1, and miR-184 together constituted a network to balance the proliferation and differentiation of NSCs.

The balance of self-renewal and differentiation of NSCs was closely related to the feedback loop formed by miR-9 and the nuclear receptor TLX [85]. The overexpression of miR-9 inhibited the proliferation of NSCs by suppressing the TLX expression, as well as accelerating neuronal differentiation. In contrast, TLX had the ability to inhibit the expression of miR-9 pri-miRNA, thereby avoiding the miR-9-induced proliferation and premature differentiation. It was found that miR-7 participated in the NSC self-renewal and differentiation by targeting Kruppel-like factor 4 (*Klf4*), a key gene for the NSC proliferation [86]. Data showed that the overexpression of miR-7 downregulated the *Klf4* gene, which in turn resulted in a decrease in the NSC proliferation and an increase in the neuronal differentiation.

MiR-124 also plays an important role in neuronal differentiation of NSCs. Six microRNAs (miR-124, miR-132, miR-134, miR-20a, miR-17-5p, and miR-30a-5p) were detected in the inner ear NSCs after 14 days of neuronal differentiation [87]. The expression of miR-124 was upregulated during neuronal differentiation, which made the tropomyosin receptor kinase B (TrkB) and the cell division control protein 42 homolog (Cdc42) upregulate, thus greatly promoting neuronal differentiation and neurite outgrowth, compared with other groups. Another team transplanted NSCs transfected with miR-124 into the spinal cord injury rats. They found that the overexpression of miR-124 increased the percentage of neurons, decreased the percentage of astrocytes, and reduced the lesion cavity volume of the spinal cord injury rats [88].

Taken together, miRNAs regulate gene expression by binding to 3′-untranslated regions (3′-UTR) of specific mRNAs [75, 89], thereby altering the proliferation and differentiation of NSCs and ultimately completing the regulation of nervous system development.

## 5. Material Factors

Material, one of the three elements of tissue engineering, plays an increasingly important role in transplanting NSCs to repair CNS injury. In recent years, with the development of tissue engineering, bio-scaffold materials which have good biocompatibility, biodegradability, three-dimensional structure, and good surface activity and are nontoxic, have effectively combined with NSCs to provide appropriate support and favorable microenvironment to enhance NSC survival, proliferation, and differentiation, so as to achieve the purpose of repairing trauma and rebuilding function. Generally, morphology, composition, and surface modification of materials are several important factors that affect cell behavior. Here, we summarized the effects of several major morphologies (film, hydrogel, and nanofiber) of different materials on the behavior of NSCs (seen in Table 3).

Film is the simplest 2D scaffold for cultured cells. Chitosan films (Chi-F), chitosan porous scaffolds (Chi-PS), and chitosan multimicrotubule conduits (Chi-MC) have been prepared to evaluate the effects on the proliferation and differentiation of NSCs. As a result, NSCs cultured on Chi-F exhibited the maximal proliferation but were more likely to differentiate into astrocytes. The proportion of neuronal differentiation of NSCs cultured on Chi-MC was the largest compared with that on Chi-F and Chi-PS [90]. By controlling the surface properties of ultrananocrystalline diamond (UNCD), the proliferation and differentiation of NSCs could be modulated [91]. The result indicated that the hydrogen-terminated UNCD film was most conducive to NSC proliferation; in addition, either the oxygen-terminated UNCD film or the hydrogen-terminated UNCD film could greatly increase the proportion of neuronal differentiation. Polyhydroxyalkanoate (PHA) is a common biopolymer material. The fusion protein PhaP-IKVAV was coated on the surfaces of two polyester materials of the same aliphatic family: poly (L-lactic acid) (PLA) and the copolymer of poly (3-hydroxybutyrate-co-3-hydroxyhexanoate) and poly (3-hydroxybutyrate-co-3-hydroxyvalerate-co-3-hydroxyhexanoate) (PHBVHHx). Ultimately, the levels of NSC adsorption and proliferation were stronger on the PHBVHHx film, whereas the neuronal differentiation and neurite outgrowth were more promoted on the PLA film [92].

Hydrogel is a network scaffold formed by the crosslinking of polymers with physical or chemical interactions. It is rich in water and has a porous structure and is conducive to material exchange, cell attachment, growth, and extension of protrusions. The gelatin-hydroxyphenylpropionic acid (Gtn-HPA) hydrogel was formed by enzyme-mediated oxidative crosslinking. The viability of NSCs encapsulated in the Gtn-HPA hydrogel was increased by approximately 8 times. Moreover, the Gtn-HPA hydrogel increased the proportion of neuronal differentiation to a greater extent compared with the blank control group [93]. Hyaluronic acid (HA) is a biocompatible and biodegradable biomaterial that is abundant in the connective tissue. The number of glial cells, neurons, or immature/progenitor cells was increased when NSCs were encapsulated into HA hydrogels and transplanted into the brain injury sites [94]. Studies have shown that polyethylene glycol (PEG) was a material with the abilities of suppressing the production of free radicals [95], inhibiting apoptotic cell death following traumatic spinal cord injury [96], supporting the repair of damaged neuronal membranes [97], and transferring oxygen and nutriments [98]. Arginyl glycyl aspartic acid (RGD) was bound to PEG to form a hydrogel that facilitated the survival, the neuronal differentiation of NSCs, and the growth and extension of neurites [99].

The electrospinning technique by which materials are made into three-dimensional nanofiber scaffolds has been used in tissue engineering. NSCs were cultured on laminin-coated electrospun polyethersulfone (PES) fiber meshes. With the decrease of fiber diameter, the level of NSC proliferation and migration increased, while the degree of cell aggregation decreased. Moreover, the differentiation of NSCs was significantly affected by the diameter of fibers [100]. Three PHA materials were selected as nanofiber scaffolds for cell culture, including poly (3-hydroxybutyrate) (PHB), copolymer of 3-hydroxybutyrate and 4-hydroxybutyrate (P3HB4HB), and copolymer of 3-hydroxybutyrate and 3-hydroxyhexanoate (PHBHHx). These three nanofiber scaffolds supported the growth and differentiation of NSCs, in which PHBHHx showed the strongest ability to promote the proliferation and neuronal differentiation of NSCs [101]. Researchers immobilized BDNF on poly-*ε*-caprolactone (PCL) nanofibers, which could support NSC proliferation and differentiation into neurons and oligodendrocytes [102] (seen in Table 3).

Artificial biomaterial scaffolds cannot only fill the tissue defects, which is conducive to the attachment, migration, and growth of endogenous and exogenous NSCs, but also regulate the microenvironment around the lesion by controlled releasing active factors so as to achieve nerve regeneration. Tissue engineering combines materials with NSCs to provide a promising approach for solving the barrier of nerve regeneration in the CNS.

## 6. Cell Signaling Pathways

The intrinsic regulatory mechanisms of the four factors discussed above affecting the behavior of NSCs may be related to the cell signaling pathways. Growing evidence has suggested that the self-renewal and differentiation of NSCs are due to the common integration of multiple cell signaling systems in the microenvironment of the cells. Consequently, elucidating the regulatory mechanisms of NSCs is critical for the study about the development of the nerve system, the repair of injured nerves, and cell transplantation for the treatment of CNS diseases. Here, we mainly focused on the mechanism of the regulation of NSCs through the Wnt and the Notch signaling pathways.

### 6.1. Wnt Signaling Pathway

The Wnt signaling pathway is a highly conserved signaling pathway in the evolution of species, which plays a vital role in the early development, organogenesis, tissue regeneration, and other physiological processes of animal embryos. The main components of the Wnt/*β*-catenin signaling pathway include the secreted protein Wnt family, the transmembrane receptor Frizzled family, low-density lipoprotein receptor-related protein (LRP), Dishevelled (Dsh), glycogen synthase kinase3*β* (GSK3*β*), Axin, *β*-catenin, adenomatous polyposis coli protein (APC), and the transcription factor T cell factor/lymphoid enhancer factor (TCF/LEF) family. When the Wnt is in a resting state, *β*-catenin, GSK3*β*, APC, and Axin constitute a degraded complex which causes the phosphorylation of *β*-catenin, the ubiquitination mediated by *β*-TrCP, and the degradation by protease eventually. When the Wnt ligand binds to the Frizzled and LRP5/6, the Wnt signaling pathway is activated to inhibit the formation of degraded complexes, reduce the activity of GSK3*β*, decrease the degradation, and increase the aggregation of *β*-catenin. Then *β*-catenin enters the nucleus and binds to the transcription factor of the TCF/LEF family to initiate the transcription of downstream target genes such as *Cyclin D1*, *neurogenin-1* (*Ngn-1*), and *Ngn-2* [103].

**Figure 1:**
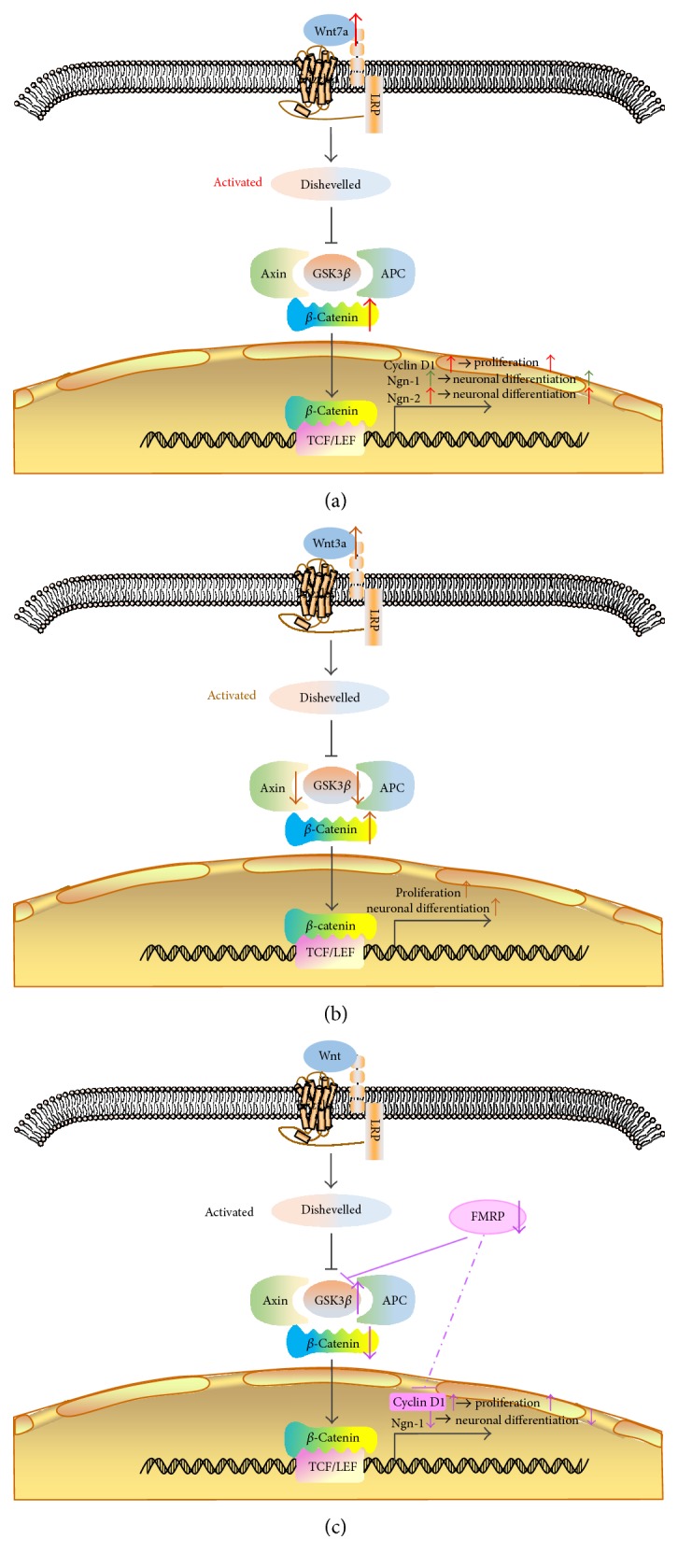
Effects of different proteins on the Wnt signaling pathway. (a) The increase in the expression of Wnt7a activated the Wnt7a/*β*-catenin-cyclinD1 pathway, which stimulated the proliferation of NSCs, and induced the Wnt7a/*β*-catenin-Ngn2 pathway, which promoted the neuronal differentiation (the red arrows). In the cerebral cortex of mice, the overexpression of Wnt7a could increase the level of Ngn-1 mRNA and then induce the differentiation of NSCs into neurons (the green arrows). (b) The overexpression of Wnt3a activated the Wnt signaling pathway and promoted the proliferation and neuronal differentiation of NSCs. (c) The loss of FMRP resulted in a decrease in the level of *β*-catenin, then downregulated the expression of *Ngn-1* and reduced neuronal differentiation.

Wnt7a is critical for self-renewal and neuronal differentiation of NSCs. In the hippocampal dentate gyrus of adult mice, a decrease in the expression of Wnt7a accelerated the withdrawal of NSCs from the cell cycle, and the proliferation of NSCs reduced significantly. Dramatically, the number of mature neurons differentiated by NSCs was also significantly reduced when the expression of Wnt7a was decreased. The study demonstrated that Wnt7a could regulate the expression of different downstream target genes in the transcriptional level to achieve the bidirectional regulation of NSC behavior. The activation of the Wnt7a/*β*-catenin-cyclinD1 pathway was able to stimulate NSC proliferation, and the activation of the Wnt7a/*β*-catenin-Ngn-2 pathway promoted neuronal differentiation [104] (seen in Figure 1(a)). At the same time, the overexpression of Wnt7a could increase the level of Ngn-1 mRNA and then induce the differentiation of NSCs into neurons in the cerebral cortex of mice [105] (seen in Figure 1(a)). Wnt3a has been reported to be involved in the survival, proliferation, and differentiation of NSCs through the Wnt/*β*-catenin pathway [106]. The feasibility of Wnt3a acting on NSCs to repair the retina has been investigated [107]. The overexpression of Wnt3a led to the instability of Axin and the inhibition of GSK3*β* activity, thereby activating the Wnt signaling pathway and ultimately promoting the proliferation and neuronal differentiation of NSCs (seen in Figure 1(b)). Fragile X mental retardation protein (FMRP) is a regulatory protein for hereditary mental retardation. The absence of FMRP caused a decrease in the level of *β*-catenin due to the disorder of GSK3*β* and then downregulated the expression of *Ngn-1* [108]. Ngn-1 was an initiator of early neuronal differentiation and an inhibitor of astrocyte differentiation [109, 110]. Eventually, the differentiation of neurons was reduced and the differentiation of glial cells was increased. In addition, the inhibition of NSC proliferation was weakened by the absence of FMRP (seen in Figure 1(c)).

### 6.2. Notch Signaling Pathway

The Notch signaling pathway is widespread and highly conserved in invertebrates and vertebrates. The core components of the classical Notch signaling pathway are mainly composed of Notch receptors (Notch1–4), Notch ligands (the Delta/Serrate/lag-2 protein (DSL), such as Jagged1, Jagged2, and Delta-like1–4), CSL (a class of DNA-binding proteins), and some regulatory molecules. When the Notch receptor binds to the ligand, the Notch intracellular domain (NICD) is released by the receptor after three times of shearing, then enters the nucleus to form a NICD/CSL transcriptional activator which activates the target genes of the basic-helix-loop-helix (bHLH) transcriptional repressor family [111]. Studies have shown that the Notch signaling pathway is one of the important signaling pathways that affect the self-renewal, differentiation, and internal stability of NSCs.

**Figure 2:**
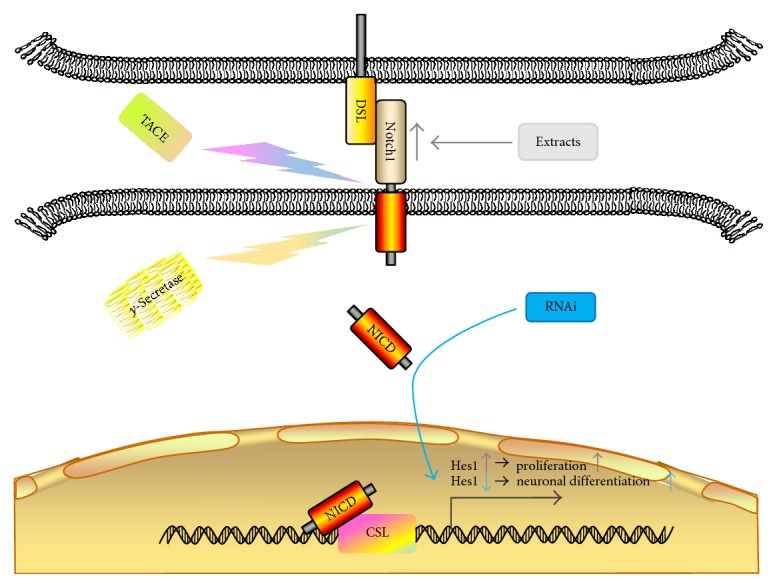
The upregulation and downregulation of *Hes1* in the Notch signaling pathway. After the TBI in mice, the RNAi downregulated the expression of *Hes1*, and the NSCs in the DG differentiated into neurons heavily (the blue arrows). The extracts from the injured spinal cord upregulated the expression of Notch1 and *Hes1*, activated the Notch signaling pathway, and promoted the proliferation of NSCs (the gray arrows).

External factors mainly mediate the differentiation inhibitory signal through the “side inhibition” mechanism of the Notch signaling pathway, which prevents the differentiation of adjacent NSCs and promotes their proliferation. Thus, the Notch signaling pathway plays a key role in the self-renewal and maintenance of NSCs during the brain development. Some researchers reported that *Hes1* could promote the proliferation and inhibit the differentiation of NSCs [112, 113]. After the traumatic brain injury (TBI) in mice, the expression of *Hes1* was downregulated by RNA interference (RNAi). The NSCs in the dentate gyrus (DG) differentiated into neurons largely, which improved the spatial learning and memory ability of the mice and further restored the neurological function [114] (seen in Figure 2). The extracts from the injured spinal cord upregulated Notch1 mRNA, and the expression of *Hes1* subsequently activated the Notch signaling pathway to promote NSC proliferation [115] (seen in Figure 2). Nevertheless, silencing the expression of *Notch1* inhibited the division of NSCs and prevented NSCs from entering the cell cycle and maintaining self-renewal.

**Figure 3:**
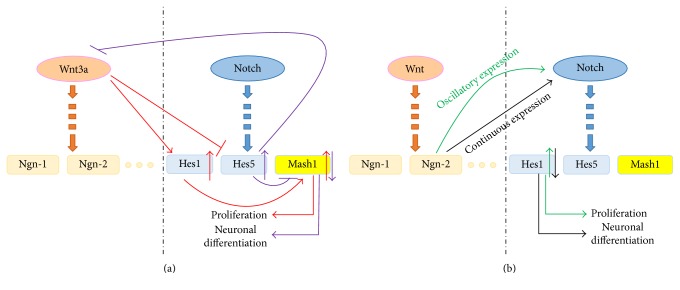
The interaction between the Wnt and the Notch signaling pathways. (a) Wnt3a could upregulate the expression of the downstream target gene *Hes1* of the Notch signaling pathway and inhibit the expression of *Hes5* and then induce the proliferation of NSCs (the red arrows). The overexpression of *Hes5* inhibited the Wnt signaling pathway, downregulated *Mash1*, and induced neuronal differentiation (the purple arrows). (b) The oscillatory expression of *Ngn-2* could activate the Notch signaling pathway so that *Hes1* was upregulated to promote the self-renewal of NSCs (the green arrows). The continuous expression of Ngn-2 inhibited the expression of *Hes1* and promoted the differentiation of NSCs into neurons (the black arrows).

Several studies have demonstrated that the Wnt and Notch signaling pathways interact with each other during NSC differentiation. Wnt3a could upregulate the downstream target gene *Hes1* of the Notch signaling pathway and continue to inhibit the expression of *Hes5*, improve the level of *Mash1*, and then induce the proliferation of NSCs. The overexpression of *Hes5* inhibited the Wnt signaling pathway, downregulated *Mash1*, and induced neuronal differentiation [116] (seen in Figure 3(a)). Besides, when the *Ngn-2* expression dynamically oscillated, it was capable of inducing the expression of Delta-like1 (Dll1) in the adjacent cells and activating the Notch signaling pathway so that *Hes1* was upregulated to promote self-renewal of NSCs; when *Ngn-2* was expressed persistently, it inhibited the *Hes1* expression and promoted the differentiation of NSCs into neurons [117, 118] (seen in Figure 3(b)).

To sum up, the proliferation and differentiation of NSCs are involved in the coordination and integration of multiple cell signaling pathways. However, the precise regulation mechanism of NSCs is still not clear.

## 7. Conclusion

In this article, we mainly summarize the factors which have been widely studied in the last decade. These different factors have different effects on the behavior of NSCs which have become the candidate for repairing CNS injury due to their ability of self-renewal and multiple differentiation. Physical factors usually modulate the behavior of NSCs by controlling specific parameters and the sites of exertion. While chemical factors affect the behavior of NSCs according to the different molecular structures and properties of different chemical molecules, biological factors, such as neurotrophins, growth factors, and microRNAs, are involved in the endogenous regulation of the NSC proliferation and differentiation. Moreover, NSCs are also subject to exogenous regulation of the microenvironment which is constituted by adjacent cells and extracellular matrices (including various proteins). Materials provide the structural support for the injured sites, which forms a favorable microenvironment, thereby promoting the proliferation and differentiation of NSCs. It is interesting that the regulation of these four factors on the NSC behavior is closely related to the expression of related genes. Therefore, we believe that the cell signaling pathway is the underlying mechanism that regulates the NSC proliferation and differentiation by different factors.

Although significant progress has been made in the treatment of CNS diseases by using NSCs, there are still many problems that need to be addressed. For example, (1) the mechanism of precise regulation of NSCs after transplantation is still unclear; (2) there may be complications of transplantation; and (3) at present, most stem cell transplantation is only in the animal experimental stage and the application of the NSC transplantation in clinical still has a great risk. Researchers have gradually realized the existence of these problems. We believe that in the near future, these problems will be solved and NSCs will play a more and more important role in treating CNS diseases.

## Figures and Tables

**Table 1:** Effects of drugs or active ingredients on the proliferation and differentiation of NSCs.

Drugs or active ingredients	Cells	The role of proliferation	The role of differentiation	Mechanism
Salvianolic acid B [21]	NSCs from cortex of E13.5 mice	Nestin-positive cells ↑	The expression of tau ↑; the expression of GFAP ↓	Cells in G2/S phase
Tetramethylpyrazine [23]	NSCs from cortex of E15 SD fetal rats	BrdU-positive cells ↑	*β*-Tubulin III-positive cells ↑; *β*-tubulin III/nestin-positive cells ↑; GFAP-positive cells ↓; GFAP/nestin-positive cells ↓	The expression of cyclin D1 ↑; the expression of P-ERK ↑; the expression of P-JNK with little change; the phosphorylation of P38 ↓
Epimedium flavonoids [24]	NSCs from hippocampus of 1d neonatal rats	BrdU-positive cells ↑	Tuj1-positive cells ↑; NF-200-positive cells ↑; GFAP-positive cells ↑	
Panax notoginseng saponins [25]	NSCs from hippocampus of 1d neonatal rats	Nestin-positive cells ↑; BrdU-positive cells ↑	Nestin/BrdU-positive cells ↑; nestin/vimentin-positive cells ↑; nestin/Tuj1-positive cells ↑	
Tenuigenin [26]	NSCs from hippocampus of E16 rats	BrdU-positive cells ↑	*β*-Tubulin III-positive cells ↑; GFAP-positive cells ↑	
Gastrodiae rhizome [27]	NSCs from human embryos	Viability ↑	The expression of nestin ↓; the expression of Tuj1 ↑; the expression of MAP2 ↑; dendrites ↑	The expression of Sox2 ↓
Astragaloside IV [28]	NSCs from hippocampus of adult SD rats	BrdU-positive cells ↑	BrdU/MAP2-positive cells ↑; BrdU/GFAP-positive cells ↑	The expression of NGF ↑
Angelica [29]	NSCs from embryonic rats	Nestin ↓ compared with the hypoxia group; nestin ↑ compared with the control group		
Ketamine [31]	NSCs from the SVZ of 7d neonatal male SD rats	Nestin/BrdU-positive cells ↓	The expression of *β*-tubulin III ↑; the expression of GFAP ↓; the expression of nestin ↓	
Fingolimod [34]	NSCs from E13.5 SD rats	CCK-8 ↑	The expression of GFAP ↑	
Simvastatin [36]	NSCs from embryonic rats	Nestin-positive cells ↑	GFAP-positive cells ↑; MAP2-positive cells ↑	The expression of Notch1 ↑

**Table 2:** Effects of microRNAs on the proliferation and differentiation of NSCs.

MicroRNA	The role of proliferation	The role of differentiation	Mechanism
miR-34a [75]	Postmitotic neurons ↑	Neurite elongation ↑	miR-34a ↑ → SIRT1 ↓ → P53 acetylation ↑
miR-125b [77]	BrdU-positive cells ↓	Nestin-positive cells ↓; Sox2-positive cells ↓; vimentin-positive cells ↓; MAP2-positive cells ↑; Tuj1-positive cells ↑; cell migration ↑	miR-125b ↑ → nestin ↓
miR-146 [81]	Neural sphere diameter ↓	*β*-Tubulin III positive cells ↓; GFAP-positive cells ↑	miR-146 ↑ → Notch1 ↓
miR-342-5p [82]	BrdU-positive cells ↓	PAX6-positive cells ↓; TBR2-positive cells ↑; GFAP-positive cells ↓	Notch → Hes5 ↓ → miR-342-5p ↑ → GFAP ↓
miR-184 [84]	BrdU-positive cells ↑	Tuj1-positive cells ↓; GFAP-positive cells ↓	MBD1 ↓ → miR-184 ↑ → Numbl ↑
miR-9 [85]	BrdU-positive cells ↓	Tuj1-positive cells ↑; GFAP-positive cells ↑	miR-9 ↑ → TLX ↓ → proliferation ↓, neurons ↑
miR-7 [86]	G1/S cell cycle arrest	Tuj1-positive cells ↑; MAP2-positive cells ↑; Nestin-positive cells ↓; vimentin-positive cells ↓	miR-7 ↑ → *Klf4* ↓ → proliferation ↓, neurons ↑
miR-124 [87, 88]	BrdU-positive cells ↓	Tuj1-positive cells ↑; NeuN-positive cells ↑; GFAP-positive cells ↓	miR-124 ↑ → TrkB ↑,Cdc42 ↑ → neurons ↑

**Table 3:** Effects of biomaterials on the proliferation and differentiation of NSCs.

Topography	Materials	Dimensions	Cells	Outcome
Film	Chitosan [90]		NSCs from cortex of E12 fetal rats	Proliferation ↑; astrocytes ↑
Film	Ultrananocrystalline diamond (UNCD) [91]		NSCs from cortex of E11.5 rat embryos	Proliferation most on hydrogen-terminated UNCD film; neuronal differentiation most on oxygen-terminated UNCD film
Film	PHA: PLA, PHBVHHx [92]		NSCs from neocortex of E13–15 rat embryos	Adsorption and proliferation more on PHBVHHx film; neuronal differentiation and neurite outgrowth more on PLA film
Hydrogel	Gelatin-hydroxyphenylpropionic acid (Gtn-HPA) [93]		NSCs from hippocampus of adult female Fischer 344 rats	Viability ↑; proliferation rate ↓; the expression of GFAP ↑; the expression of Tuj-1 ↑
Hydrogel	Hyaluronic acid (HA) [94]		NSCs from induced pluripotent stem cells	Glial, neuronal, or immature/progenitor states ↑; proliferation ↑
Hydrogel	Polyethylene glycol [99]		NSCs from the BMSCs of 4 weeks SD rats	Viability ↑; proliferation ↑ slightly; neuron ↑; neurite outgrowth and extension
Nanofibers	Polyethersulfone (PES) [100]	283 nm, 749 nm, 1452 nm	NSCs from hippocampus of adult rats	40% ↑ in oligodendrocyte with 283 nm fibers; 20% ↑ in neuronal cells with 749 nm fibers; proliferation ↑; cell spreading ↑; cell aggregation ↓ with the decrease of fiber diameter
Nanofibers	Polyhydroxyalkanoates (PHA): PHB, P3HB4HB, PHBHHx [101]		NSCs from neocortex of E13–15 rat embryos	Proliferation and neuronal differentiation most in PHBHHx
Nanofibers	Poly-*ε*-caprolactone (PCL) [102]	550 ± 100 nm	NSCs from neocortex of E14.5 rat embryos	Proliferation ↑; neurons ↑; oligodendrocytes ↑
